# Three-dimensional diffraction mapping by tuning the X-ray energy

**DOI:** 10.1107/S0909049511003190

**Published:** 2011-03-15

**Authors:** T. W. Cornelius, D. Carbone, V. L. R. Jacques, T. U. Schülli, T. H. Metzger

**Affiliations:** a European Synchrotron Radiation Facility, 6 rue Jules Horowitz, BP 220, 38043 Grenoble Cedex, France; b Max Planck Institute of Colloids and Interfaces, 14424 Potsdam, Germany

**Keywords:** X-ray diffraction, nanofocused and microfocused XRD, energy scan, three-dimensional reciprocal-space mapping

## Abstract

Three-dimensional reciprocal-space maps of a single SiGe island around the Si(004) Bragg peak are recorded using an energy-tuning technique with a microfocused X-ray beam with compound refractive lenses as focusing optics. The map is in agreement with simulated data as well as with a map recorded by an ordinary rocking-curve scan. The energy-tuning approach circumvents both the comparatively large sphere of confusion of diffractometers compared with nanostructures and vibrations induced by motors. Thus, this method offers new possibilities for novel combinations of three-dimensional micro- and nano-focused X-ray diffraction with complex *in situ* sample environments such as scanning probe microscopes.

## Abstract 2

Three-dimensional reciprocal-space maps of a single SiGe island around the Si(004) Bragg peak are recorded using an energy-tuning technique with a microfocused X-ray beam with compound refractive lenses as focusing optics.

## 1. Introduction

Nanomaterials have been attracting enormous attention for the past few years owing to the influence of the spatial confinement on their physical properties such as the density of states, the band structure and the mechanics. For their structural characterization numerous methods are employed such as scanning and transmission electron microscopy (SEM and TEM) or X-ray diffraction (XRD). The preparation of electron-transparent samples for TEM analysis could damage the nanostructures whereas XRD is basically a non-invasive technique. Beam damage is mostly an issue for soft condensed matter and biological samples while hard condensed matter is rather resistant to the radiation. In recent years the use of X-ray micro- and nano-beams as local probes for the investigation of single nanostructures has increased at synchrotrons. This is due to the increased lateral resolution along with the increased photon flux density (Mocuta *et al.*, 2008 ▶). X-rays can be focused by reflective, refractive and diffractive optics. The first group includes achromatic focusing optics such as Kirkpatrick–Baez (KB) mirrors or waveguides (Kirkpatrick & Baez, 1948 ▶; Bilderback *et al.*, 1994 ▶). For chromatic-type optics, belonging to the second group, *e.g.* compound refractive lenses (CRLs) and Fresnel zones plates, the focal length depends on the X-ray energy (Snigirev *et al.*, 1996 ▶; David *et al.*, 2000 ▶). Focal spot sizes of a few hundred nanometers are routinely obtained rendering it possible to study single nanostructures. Focused X-ray beams are used in various scattering experiments including SAXS or Bragg geometry for the characterization of morphology and strain, respectively. The possibility to generate coherent focused beams triggered the development of coherent scattering techniques which are now commonly applied (Sutton *et al.*, 1991 ▶; Robinson & Harder, 2009 ▶; Diaz *et al.*, 2009 ▶; Schroer *et al.*, 2008 ▶; Favre-Nicolin *et al.*, 2009 ▶; Chamard *et al.*, 2010 ▶).

In this manuscript we focus on the use of nanobeams for XRD experiments. Owing to the extension of the diffracted signal from a nanostructure (form factor and strain) in reciprocal space, area detectors are generally used to efficiently record intensity. However, the two-dimensional XRD images represent one specific cut through reciprocal space. Owing to its small size and an eventually inhomogeneous strain distribution, a nanostructure is expected to exhibit an extended three-dimensional diffraction pattern. In Bragg geometry, three-dimensional intensity distributions are obtained by performing rocking scans of a few degrees through the selected Bragg peak and simultaneously recording two-dimensional intensity cuts (Fewster, 1997 ▶). One main issue concerning the collection of three-dimensional diffraction data is the large sphere of confusion of existing diffractometers (typically a few tens of micrometers over a 360° rotation) as compared with the sample and beam size (a few hundreds of nanometers). Even though the rocking curve is taken over typically 1° only, the investigated nanostructure may move out of the beam. In the special case of coherent diffraction even a movement of the sample within the beam focus may be detrimental since different parts of the beam having slightly different wavefronts are diffracted. This variation in the wavefront may complicate the inversion of the coherent image. The acquisition of three-dimensional patterns thus requires the re-alignment of the structure in the nano-beam for every rocking angle, resulting in an increase of the measuring time. Furthermore, the presence of complex sample environments for *in situ* XRD measurements may demand a limitation of sample movement to reduce or avoid any vibrations induced by the diffractometer movement. As an example, the *in situ* atomic force microscope (AFM) available at the ID01 beamline (ESRF, Grenoble) is used for *in situ* compression tests to study the mechanical properties of single nanostructures (Scheler *et al.*, 2009 ▶; Rodrigues *et al.*, 2009 ▶). If the AFM tip is in contact with the nanostructure under investigation, any movement or vibration must be avoided to prevent the sample surface or the AFM tip from being damaged when rocking the sample. Therefore, a method of recording three-dimensional reciprocal-space maps (3D-RSMs) without moving the sample is highly desirable. Here, we present how this can be achieved by scanning the X-ray energy. The energy of the incident photons is varied in a pre-defined range and two-dimensional XRD images are taken at each energy step. This method avoids any movements of the diffractometer motors. For the proposed approach the use of *achromatic* focusing optics such as KB mirrors is an obvious choice. However, and most interestingly, we demonstrate here that *chromatic* optics (namely Be CRLs) can be successfully used for this purpose, at the expense of an increase in measurement time. The resulting three-dimensional map is compared with the same map obtained by the classical approach of rocking the Bragg angle.

## 2. Experimental method

**Figure 1:**
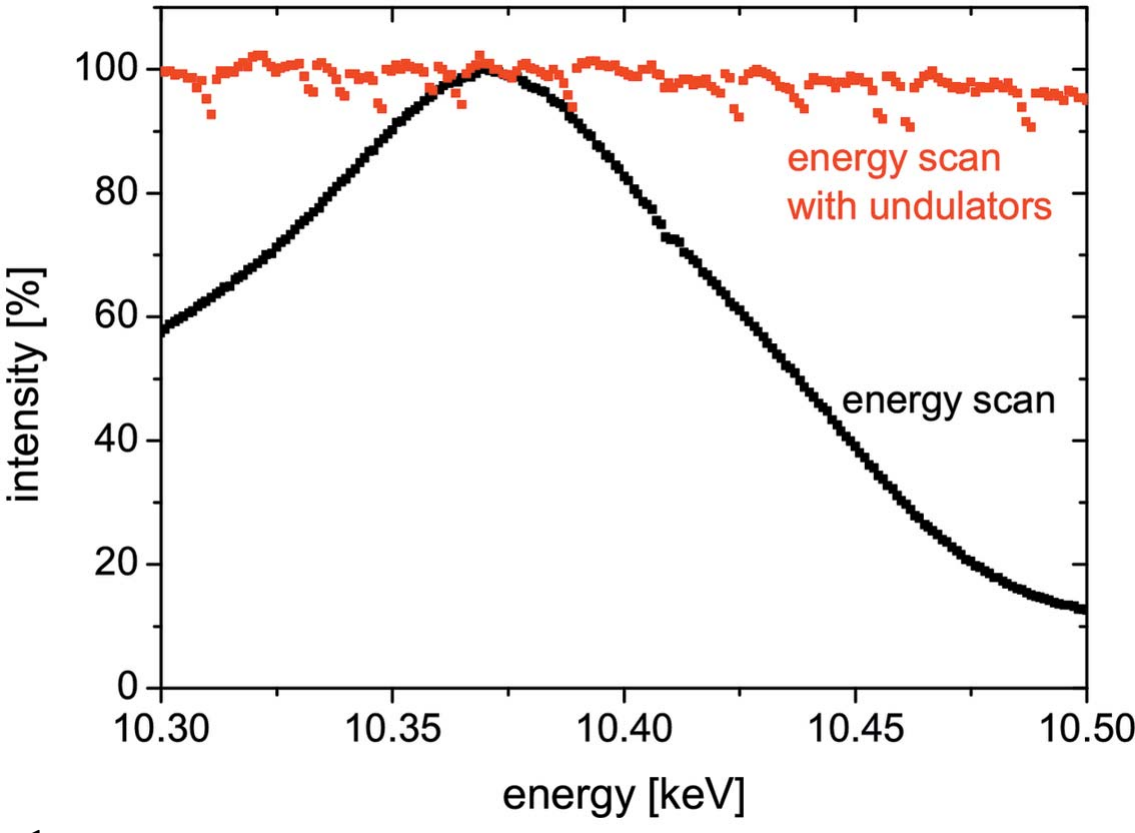
Normalized incident intensity as a function of energy with and without adjusting the undulator gap. The intensity fluctuations for the energy scan with undulator gap adjustment originate from the positioning accuracy of the undulator gap.

For demonstration purposes we measure the 3D-RSM of a self-assembled SiGe(001) island grown epitaxically on a Si(001) substrate by liquid-phase epitaxy. The island has the shape of a truncated pyramid with a base width of 1 µm, a height of 500 nm and a 300 nm-sized top (001) facet. The X-rays are monochromated by a double-bounce Si(111) channel-cut monochromator at an energy of 10.4 keV. In order to record the 3D-RSM the energy is varied by Δ*E* ± 100 eV in steps of 1 eV corresponding to a variation of Δ*Q* = ±0.475 nm^−1^. While scanning the energy the undulator gap is adjusted to stay on the maximum of the undulator emission peak keeping the incident intensity constant. Fig. 1 ▶ displays the intensity as a function of the X-ray energy with and without re-adjustment of the undulator gap. While the intensity stays fairly constant with the gap adjustment, the intensity drops by ∼90% without adjustment. The intensity fluctuations for the energy scan with undulator gap adjustment probably originates from the positioning accuracy of the undulator gap.

**Figure 2:**
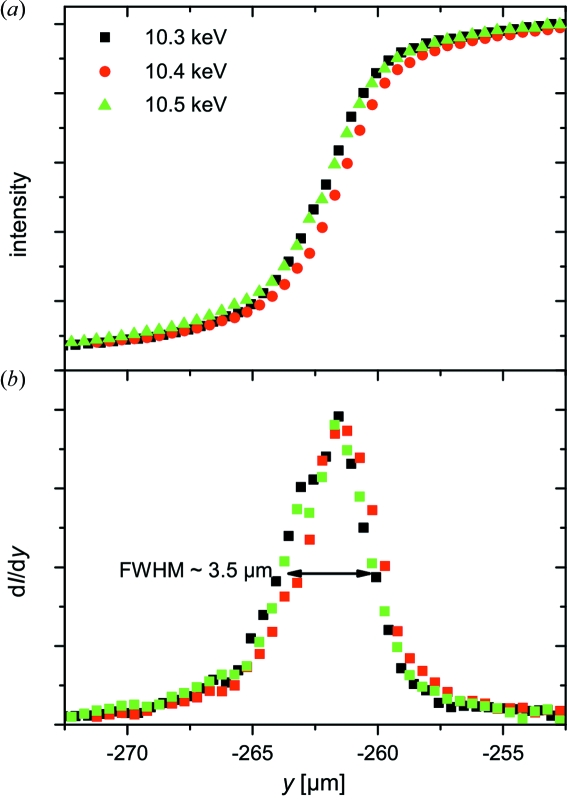
(*a*) Normalized intensity during the ‘knife-edge scans’ of a 250 µm-thick tantalum wire at 10.3, 10.4 and 10.5 keV. (*b*) Derivatives of the intensity during the knife-edge scans shown in (*a*). The full width at half-maximum (FWHM) represents a horizontal focal size of ∼3.5 µm.

The X-ray beam is focused employing Be CRLs whose focal length *f* depends on the square of the X-ray energy (Aristov *et al.*, 2000 ▶),
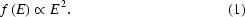
Specifically, we use 37 lenses with *f* = 90 cm at 10.5 keV. During the energy scan the CRL sample distance was adjusted according to *f*(*E*). Note that the parallelism of the lenses with respect to the X-ray beam was aligned after each translation. Both the size and the position of the focal spot at different energies were determined by ‘knife-edge scans’. For this purpose a 250 µm-thick tantalum wire was scanned through the X-ray beam while the absorption was recorded. Fig. 2(*a*) ▶ displays three horizontal knife-edge scans at 10.3, 10.4 and 10.5 keV where the focal distance of the CRLs was adjusted for each energy. The variation of the focal size and of the position of the focal spot are negligible in comparison with both the sample and the beam size (Fig. 2*b* ▶). The latter was determined by the slope of the curve obtained by the knife-edge scan. The measured focal spot size was ∼3.5 and ∼2.5 µm in the horizontal and vertical direction, respectively.

The X-ray intensity around the SiGe(004) reflection was recorded using a MAXIPIX (Ponchut *et al.*, 2007 ▶) two-dimensional detector with 256 × 256 pixels of size 55 µm × 55 µm, mounted at a distance of ∼1 m from the sample.

## 3. Results

**Figure 3:**
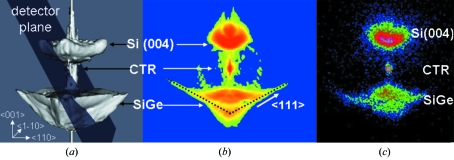
(*a*) Simulated three-dimensional reciprocal-space map of a SiGe island on Si(001) showing the diffuse scattering close to the Si(004) Bragg peak, the substrate crystal truncation rod, and the signal of the SiGe island including the CTRs of the (111) side facets of the pyramid. The semi-transparent plane represents the projection of the two-dimensional detector in the reciprocal space at the measured Bragg angle. (*b*) Simulated and (*c*) experimentally measured two-dimensional diffraction map showing similar features.

The sample topography was imaged by scanning X-ray diffraction microscopy (Mocuta *et al.*, 2008 ▶) and one specific SiGe island was placed in the microfocused beam. Fig. 3(*a*) ▶ displays the simulated 3D-RSM for a SiGe island modelled by finite-element method simulations and fast-Fourier transformations. The diffraction signal includes the Si(004) Bragg peak, the crystal truncation rod (CTR) of the Si substrate, and the signal of the SiGe island including the CTRs originating from the (111) side facets of the truncated pyramid. The semi-transparent plane represents the detector plane cutting through reciprocal space. Figs. 3(*b*) and 3(*c*) ▶ display the simulated intensity in this plane and the corresponding experimentally recorded diffraction pattern, respectively. Both images show the same features, namely the diffuse scattering close to the Si(004) Bragg reflection, the substrate CTR, and the island-induced diffuse signal at lower *Q*_*z*_ values.

**Figure 4:**
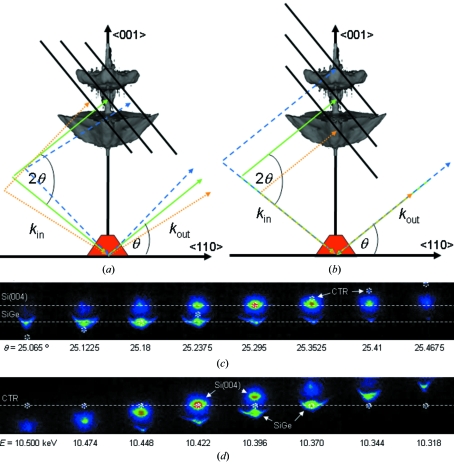
Schematics of the detector movement in reciprocal space for (*a*) a rocking-curve scan and (*b*) an energy scan. The dashed and dotted lines indicate the variation of the incident and diffracted beams. Corresponding sequences of two-dimensional XRD patterns recorded during (*c*) a rocking-curve scan and (*d*) an energy scan. To highlight the respective movement the position of the CTR is marked by circles for both cases.

Schematics of the scattering geometry for the two types of measurements of the 3D-RSM, *i.e.* rocking curve and energy tuning, are presented in Figs. 4(*a*) and 4(*b*) ▶, respectively. The dashed and dotted lines indicate the variation of the wavevector for the incident *k*_in_ and the diffracted *k*_out_ beam while the solid black lines illustrate the movement of the two-dimensional detector in reciprocal space when recording the three-dimensional intensity distribution. For a rocking-scan type of measurement the SiGe(004) Bragg reflection stays at the same position on the detector while the CTR signal moves. In contrast, during an energy scan the CTR is expected to remain at the same position on the detector, while the Bragg reflection moves as in a θ–2θ scan (radial scan). Sequences of XRD images recorded while rocking the sample in an angular range of Δθ = ± 0.5° (Δ*Q* = ±0.82 nm^−1^) and tuning the energy from 10.5 to 10.3 keV (Δ*Q* = ±0.475 nm^−1^) are presented in Figs. 4(*c*) and 4(*d*) ▶, respectively, showing the features described above. In both sequences the CTR signal is encircled to highlight its position in the two-dimensional cuts through the two types of RSM scans.

**Figure 5:**
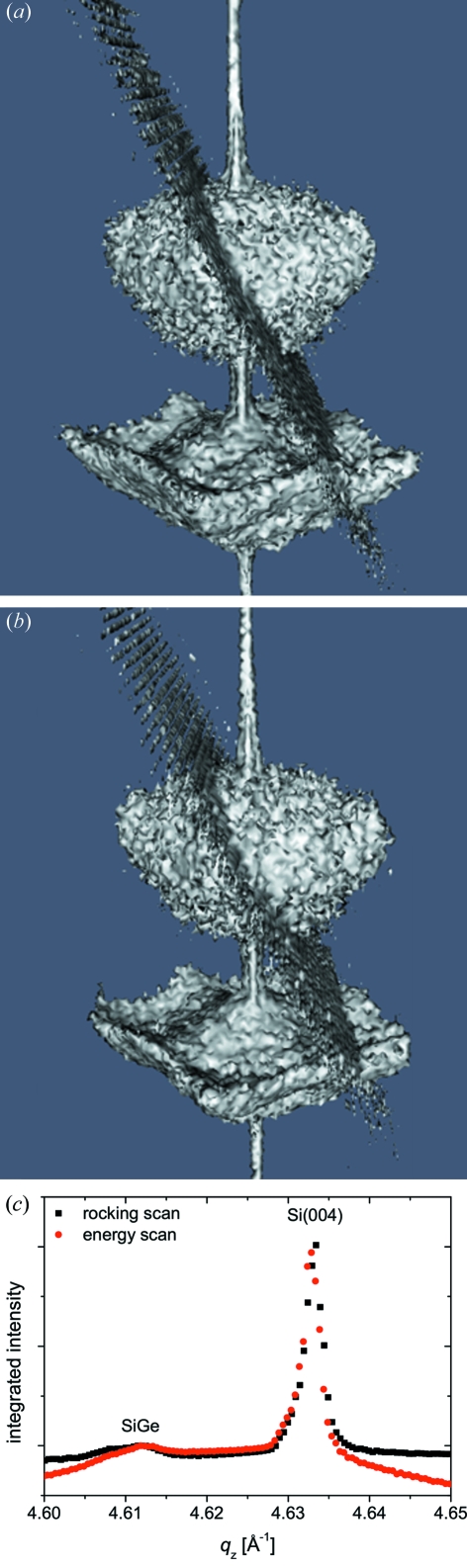
Three-dimensional reciprocal-space maps in *Q* space reconstructed from (*a*) a rocking-curve scan and (*b*) an energy scan. (*c*) *Q*_*z*_ profiles of the two 3D-RSMs shown in (*a*) and (*b*).

The three-dimensional intensity distributions in *Q* space reconstructed from the rocking-curve scan and the energy-tuning scan, from image sequences like the one displayed in Fig. 4 ▶, are presented in Figs. 5(*a*) and 5(*b*) ▶, respectively. Both reconstructions show the same features and they are in agreement with the simulated three-dimensional intensity distribution (see Fig. 3*a* ▶). The *Q*_*z*_ profiles of the two 3D-RSMs displayed in Fig. 5(*c*) ▶ prove that both approaches give the same result with very similar signal-to-noise ratios. In order to obtain the same resolution as for ordinary rocking-curve scans an accuracy of 1–2 eV is necessary. This is routinely achieved at our beamline. The Bragg reflection of the SiGe island occurs at *Q*_*z*_ = 4.612 Å^−1^ while the Si(004) Bragg peak is situated at *Q*_*z*_ = 4.633 Å^−1^. Thus the atomic lattice of the SiGe island is stretched by 0.45% compared with that of pure Si.

The intensity plane cutting through the Si(004) Bragg peak is most probably caused by air scattering and small-angle scattering from the Be lenses. A part of the X-rays diffusely scattered in front of the sample exhibit the correct incident angle on the substrate fulfilling the Bragg condition and, thus, they are reflected to the detector. The amount of such unwanted diffuse scattering contributions and, hence, the parasitic intensity plane can be reduced by inserting an aperture close to the sample position (not done here).

Rocking the sample at a fixed energy is indeed the fastest and easiest way for obtaining a three-dimensional intensity map while additional alignments are necessary for energy scans with *chromatic* optics. Hence, this approach is only possible at the expense of an increase of measurement time from a few minutes for a rocking-curve scan to about two hours for an energy scan. When the sample is re-aligned during the rocking-curve scan the experimental time increases up to half an hour depending on how often this adjustment is repeated. The preliminary tests to evaluate the lens–sample distance for different energies also require an additional time of about one hour, but they are done once for all before the experiment. A possible drawback of the use of KB mirrors is their long-term instability, as any temperature fluctuations lead to a displacement and change of curvature of the mirrors and, thus, to a defocusing and a corresponding movement of the focal spot. CRLs, even if more complicated to use and to align, offer a better stability and a reliable beam focus, which is mandatory for a reliable quantitative measurement of the three-dimensional RSM from a nanostructure in the energy-tuning approach.

## 4. Conclusions

The energy-tuning approach with microfocused X-ray beams using *chromatic* in-line focusing optics allows for the reconstruction of the three-dimensional intensity distribution of single nanostructures. Here, we demonstrate this method by recording 3D-RSMs of individual SiGe islands close to the (004) Bragg peak, which are in excellent agreement with three-dimensional maps taken by ordinary rocking-curve scans. This technique opens the door to novel combinations of three-dimensional micro- and nano-focused X-ray diffraction with complex *in situ* sample environments such as scanning probe microscopes both preventing vibrations induced by the diffractometer motors and circumventing the limitations owing to a large diffractometer sphere of confusion.
